# Multivariate Assessment of Microbiological and Incubation Data from an Experimental Trial Evaluating Essential Oil–Based Sanitizers and Formaldehyde on Hatching Eggs

**DOI:** 10.3390/pathogens15040426

**Published:** 2026-04-15

**Authors:** Vinícius Machado dos Santos, Gabriel da Silva Oliveira, Concepta McManus

**Affiliations:** 1 Laboratory of Poultry Science, Federal Institute of Brasília—Campus Planaltina, Brasília 73380-900, Brazil; 2 Faculty of Agronomy and Veterinary Medicine, University of Brasília, Brasília 70910-900, Brazil; 3 Faculty of Veterinary Medicine and Animal Science, University of São Paulo, São Paulo 05508-270, Brazil; 4 Center for Nuclear Energy in Agriculture (CENA), University of São Paulo, São Paulo 13416-000, Brazil

**Keywords:** correlation analysis, eggshells, egg sanitization, embryonic mortality, hatchability, natural products, poultry production, sanitizers, yolk sac contamination

## Abstract

Sanitization of hatching eggs is part of established poultry management practices, and its effectiveness is essential for productive success. This study aimed to investigate the relationships between microbiological and incubation performance variables obtained from a controlled experimental dataset of hatching eggs subjected to sanitization with essential oils or not under commercial conditions, and to determine the efficacy of these sanitizers, using a multivariate approach. Data were analyzed using principal component, canonical, cluster, and discriminant analysis. The results suggested that bacterial contamination of the eggshell may promote internal contamination, leading to embryonic mortality. Essential oil-based treatments are associated with lower microbial indicators and improved hatchability, while formaldehyde showed an opposite trend despite its antibacterial efficacy. Multivariate analyses clarified the interrelationships between microbiological and incubation performance variables, allowing the identification of response patterns that evidenced the functional efficiency of essential oil–based treatments for hatching egg sanitization under commercial conditions.

## 1. Introduction

Hatching egg viability can be compromised by intrinsic breeder characteristics (e.g., age, diet, and rearing conditions) and by storage and incubation conditions (e.g., temperature, relative humidity, turning frequency, and ventilation). In addition, microbiological factors, such as bacterial contamination, may also affect this viability [1]. Thus, the poultry management system must ensure conditions that keep eggs protected from pathogenic bacteria, with intact shells and without residues. These conditions include maintaining poultry health, frequently sanitizing nests, and regularly collecting eggs. Eggs collected from the floor require special attention due to their higher risk of contamination. Regardless of their origin, all hatching eggs that are visually suitable for incubation should be sanitized after collection [2,3]. Depending on the protocol employed, decontamination of the eggshell surface can still result in embryonic infection, toxic effects, and mortality caused by both factors [4]. Therefore, sanitization cannot be considered a completely protective measure for embryonic development.

The sanitization of hatching eggs is one of the main factors influencing incubation outcomes, potentially producing positive, negative, or neutral effects on embryonic development and hatchability [5,6,7]. Currently, sanitization protocols aim not only to maximize hatch rates but also to preserve egg quality and reduce bacterial loads on the eggshell and embryonic infection [8,9,10]. This precaution is crucial, as eggs contaminated with pathogenic bacteria may produce chicks that appear healthy at hatching but later disseminate microorganisms in the environment or die within the first days of life. Despite the importance of this topic and the need to understand how different factors interact to optimize productive performance, studies directly correlating the sanitization process of hatching eggs with microbiological and incubation parameters through multivariate analysis remain limited.

The commercial sanitization of hatching eggs still raises concerns due to the toxicity of commonly used synthetic chemical sanitizers, such as formaldehyde and ozone, whose adverse effects extend beyond risks to human health. Factors such as the potential for eggshell structural failure and undesirable damage to the embryo have motivated the search for natural products for this purpose [4,11,12,13]. Many of these products, such as essential oils, exhibit in vitro antimicrobial activity against Gram-negative and Gram-positive bacteria and belong to the group of compounds with easy commercial availability. A distinctive feature of some essential oils is that, in addition to their in vitro benefits and accessibility, there are studies demonstrating their efficacy and safety under in vivo conditions in the poultry context, including in animal feed or through direct application to eggs or meat, as exemplified by the essential oils *Zingiber officinale*, *Cymbopogon flexuosus*, and *Rosmarinus officinalis* [14,15,16]. This makes these oils a priority for testing as sanitizers for hatching eggs [17].

Therefore, the present study proposes a multivariate approach to explore interrelationships among microbiological and incubation performance variables obtained from a controlled experimental dataset of hatching eggs subjected to sanitization with essential oils *Zingiber officinale*, *Cymbopogon flexuosus*, and *Rosmarinus officinalis* or not under commercial conditions, and to determine the efficacy of these sanitizers. By applying principal component, canonical, and cluster analyses, this study aims to (I) identify patterns and correlations between contamination parameters and incubation performance, (II) determine which variables most strongly influence hatchability outcomes, (III) examine the relationships between these variables and the different treatments applied, and (IV) group treatments according to their overall sanitization response profile.

## 2. Materials and Methods

### 2.1. Data Source

**Figure 1:**
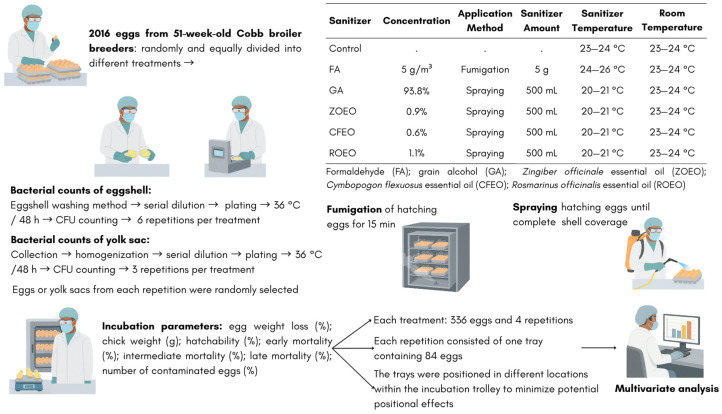
Schematic representation of the experimental process, showing the sanitization treatments and the subsequent microbiological and incubation assessments.

A pre-existing experimental dataset was used in this study. The data originated from a controlled trial conducted under commercial hatchery conditions to evaluate the effects of different sanitization treatments applied to hatching eggs after collection [17]. The experiment included hatching eggs from 51-week-old Cobb broiler breeders, randomly allocated to six treatments. Treatments involved fumigation with formaldehyde or spraying procedures using essential oil–based sanitizers. Microbiological assessments, including eggshell and yolk sac bacterial counts, as well as incubation performance parameters such as egg weight loss (%), chick weight (g), hatchability (%), early, intermediate, and late embryonic mortality (%), and the number of contaminated eggs (%), were evaluated. A schematic overview of the experimental design is presented in Figure 1.

Mean microbiological and incubation results for the different sanitization strategies are shown in Table 1. Data were analyzed using SAS software version 9.4 (SAS Institute Inc., Cary, NC, USA), with significance set at *p* < 0.05. Group comparisons were performed by ANOVA followed by Tukey’s test or by the Kruskal–Wallis test, according to data distribution. Significant differences were observed among treatments regarding chick weight and in eggshell and yolk sac mesophilic counts. Treatments based on essential oils exhibited lower bacterial counts compared to the control, whereas formaldehyde effectively reduced eggshell bacterial counts but did not decrease yolk sac counts relative to the control. Most incubation performance variables did not differ significantly among treatments. However, hatchability tended to be higher in the essential oil group [17].

### 2.2. Multivariate Analysis

Applying a multivariate analysis to this dataset is indispensable, as the effects of the sanitizers are not unidimensional. The success of sanitization depends on achieving a balance between effective microbial control and embryonic safety, requiring a statistical approach capable of integrating multiple microbiological and productive responses. This type of analysis provides a comprehensive understanding of the system, revealing how variables interrelate and influence one another, and identifying which parameters most accurately explain the differences observed among treatments.

Therefore, multivariate statistical techniques, including principal component (PROC PRINCOMP), canonical (PROC CANCORR), cluster (PROC CLUSTER), and discriminant analyses (PROC STEPDISC and PROC DISCRIM), were performed using SAS software (version 9.4; SAS Institute Inc., Cary, NC, USA). Canonical analysis was used to evaluate relationships among treatments, sets of microbiological variables, and incubation outcomes. Hierarchical clustering was applied to group treatments based on their multivariate similarity, and subsequent discriminant analyses identified the variables that most effectively differentiated the treatments. Graphical representations were generated using Microsoft^®^ Excel^®^ (Microsoft 365).

## 3. Results and Discussion

Based on the correlation analysis, the hatchability showed negative correlations with early mortality (r = −0.52, *p* < 0.05), late mortality (r = −0.57, *p* < 0.05), and the number of contaminated eggs (r = −0.41, *p* < 0.05) (Table 2). The latter also showed a positive correlation with early mortality (r = 0.39). In addition, eggshell mesophilic counts were positively correlated with yolk sac mesophilic counts (r = 0.45), while yolk sac mesophilic counts showed a negative correlation with hatchability (r = −0.34). These results indicate that increases in mortality at the beginning and end of embryonic development are associated with reduced hatchability. Likewise, a higher number of contaminated eggs tends to reduce hatchability, possibly because early mortality increases due to bacterial contamination. Furthermore, the data indicate that bacterial contamination of the eggshell contributes to contamination of the yolk sac, which may consequently reduce hatchability. A similar pattern was demonstrated by Oliveira et al. [1], who found a strong association between eggshell and yolk sac mesophilic counts (r = 0.76), as well as between eggshell mesophilic counts and yolk sac Enterobacteriaceae counts (r = 0.73). According to these authors, yolk sac Enterobacteriaceae counts were the main factor positively correlated with early mortality (r = 0.35).

### 3.1. Principal Component Analysis

**Figure 2:**
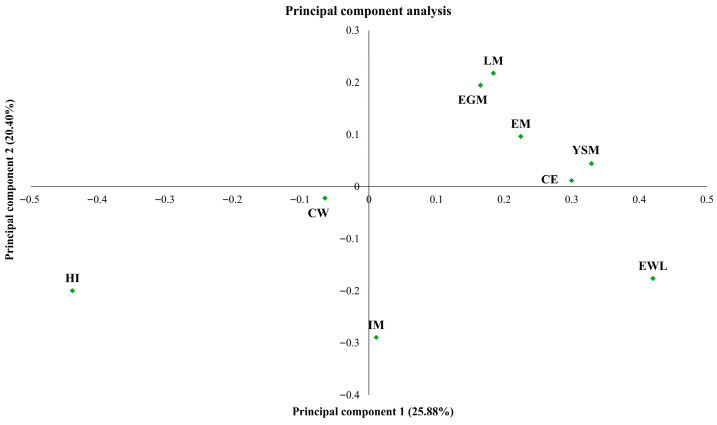
Principal component analysis showing the relationship between microbiological and incubation performance variables. Egg weight loss (EWL); chick weight (CW); Hatchability of fertile eggs (HI); early mortality (EM); intermediate mortality (IM); late mortality (LM); number of contaminated eggs (CE); eggshell mesophilic counts (EGM); yolk sac mesophilic counts (YSM).

The principal component analysis revealed a separation between variables associated with incubation performance and those related to microbial contamination (Figure 2). The first two principal components together explained 46.28% of the total data variability (25.88% for PC1 and 20.40% for PC2), reflecting the interaction between microbiological and incubation parameters. The remaining variability is distributed among the subsequent components, which capture additional, though less dominant, covariance patterns among the analyzed variables. Thus, the first two axes summarize the main gradients of association in the dataset, while higher-order components represent more subtle multivariate structures.

The variable positions indicate that microbiological factors (eggshell and yolk sac mesophilic counts and the number of contaminated eggs) exert a strong negative influence on hatchability (Figure 2). The aggregation of early mortality, number of contaminated eggs, eggshell mesophilic counts, and yolk sac mesophilic counts in the same region of the factorial plane suggests that eggshell contamination, associated with a failure in the natural or artificial antibacterial barrier, favors bacterial penetration, resulting in yolk sac contamination and embryonic mortality at the early developmental stages. Moreover, the variables late mortality and eggshell mesophilic counts, located in the upper quadrant, showed a positive association, suggesting that bacterial contamination of the eggshell may promote persistent internal contamination leading to embryonic mortality at the late developmental stages. These relationships among variables explain why sanitizing hatching eggs is an effective strategy for minimizing embryonic mortality and increasing hatchability. In the study by El-Kashef and El Sabry [18], a direct influence of eggshell contamination rate and pre-incubation sanitization strategy on embryonic mortality was observed. Bacteriological evaluations conducted at 7 and 14 days of incubation demonstrated that garlic and ginger solutions at concentrations of 1 and 2 mL/L significantly reduced the total bacterial count on the eggshell surface. Consequently, eggs with adequately sanitized shells exhibited lower embryonic mortality and higher hatchability than those without sanitization.

### 3.2. Canonical Analysis

**Figure 3:**
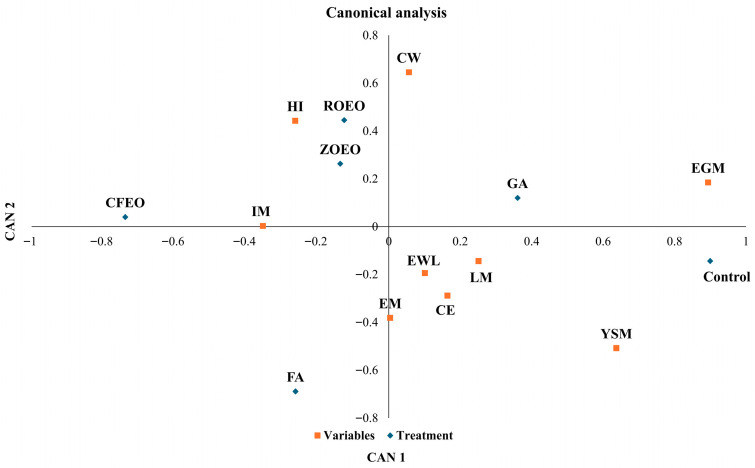
Canonical analysis showing the relationships between egg sanitization treatments and microbiological and incubation variables. Egg weight loss (EWL); chick weight (CW); Hatchability of fertile eggs (HI); early mortality (EM); intermediate mortality (IM); late mortality (LM); number of contaminated eggs (CE); eggshell mesophiles (EGM); yolk sac mesophilic counts (YSM). Grain alcohol (GA); formaldehyde (FA); *Zingiber officinale* essential oil (ZOEO); *Cymbopogon flexuosus* essential oil (CFEO); *Rosmarinus officinalis* essential oil (ROEO).

The canonical analysis revealed distinct relationships between the egg sanitization products and the response variables evaluated during incubation (Figure 3). The only treatments that showed a positive association with any microbiological variable (eggshell yolk sac mesophilic counts or the number of contaminated eggs) were the control and grain alcohol, indicating greater microbial persistence in the absence of treatment or when the method is ineffective. Treatments with formaldehyde and essential oils showed trends opposite those of the contamination variables, demonstrating antimicrobial efficacy when used for egg sanitization. Essential oil–based treatments clustered near the hatchability variable, suggesting that these formulations were beneficial to embryonic development. Formaldehyde showed an opposite trend to hatchability, suggesting a reduction in hatchability despite its proven antibacterial activity. This result reinforces the hypothesis of embryotoxicity associated with the use of formaldehyde during egg sanitization. All results are supported by the data presented in Table 1.

The literature indicates that different sanitizing agents may reduce microbial contamination on eggs but may have distinct effects on incubation performance [19,20]. Formaldehyde, despite its well-established antibacterial activity, is highly toxic and has been associated with impairments in hatchability and embryonic development [21,22]. In contrast, essential oils have been highlighted as promising alternatives, as they combine antimicrobial efficacy with lower embryotoxic potential, resulting in positive effects on variables such as hatchability and embryonic health [21,23].

Despite the promising results, the adoption of essential oils as sanitizers requires careful evaluation regarding their suitability for commercial use. Factors such as production or acquisition cost, chemical stability, volatility, compositional variability, and commercial availability may directly influence the standardization, efficiency, and economic feasibility of these formulations on a large scale. In addition, the development of sanitizer formulations involving these compounds requires specific attention to several factors, such as the concentration and the type of diluent used, in order to minimize undesirable interactions with hatching eggs.

### 3.3. Discriminant Analysis

The discriminant analysis among treatments indicated that eggshell mesophilic counts were the main variable responsible for treatment separation, explaining 84.64% of the discriminant variability (partial R^2^ = 0.8464; *p* = 0.0002) (Table 3). Subsequently, chick weight contributed 64.40% of the residual variability among treatments (partial R^2^ = 0.6440; *p* = 0.0263), while yolk sac mesophilic counts accounted for 53.48% of the additional discrimination (partial R^2^ = 0.5348). These results demonstrate that the differences observed among treatments are associated with the combined effect of microbiological variables and incubation-related outcomes. For example, the data presented in Table 1 indicate that these were the only variables analyzed that showed significant effects in response to the treatments, which explains their significant contribution to separating the treatments.

The efficiency of the sanitizing agent determines the number of bacteria surviving on the egg surface. Effective sanitization results in a significant reduction in bacterial load, whereas inefficient sanitization may allow bacterial persistence or even an increase in microbial levels. Higher bacterial loads increase the likelihood of bacterial penetration through the eggshell [24] and, consequently, pose greater risks to embryonic survival, negatively affecting productive parameters such as hatchability. Thus, eggshell mesophilic counts, identified as the main discriminant variable, constitute a key factor in explaining the differences observed among treatments and their effects on incubation outcomes.

### 3.4. Hierarchical Cluster Analysis

**Figure 4:**
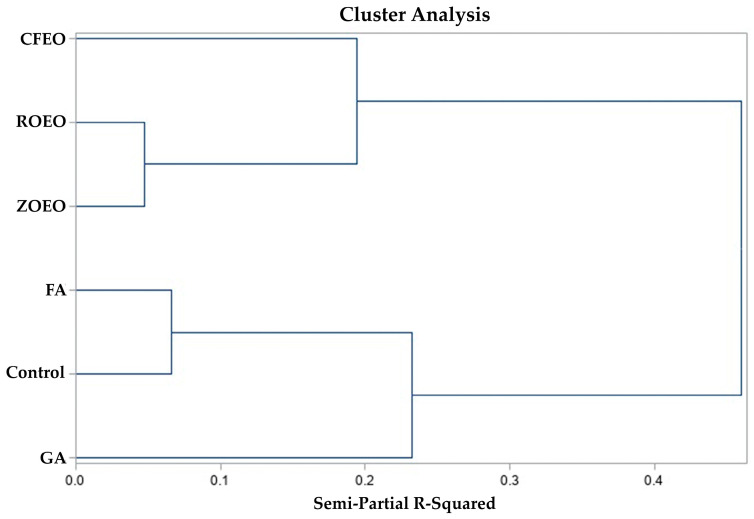
Hierarchical cluster analysis of the parameters measured during the experimental period. Grain alcohol (GA); formaldehyde (FA); *Zingiber officinale* essential oil (ZOEO); *Cymbopogon flexuosus* essential oil (CFEO); *Rosmarinus officinalis* essential oil (ROEO).

The multivariate cluster analysis supports the idea that treatments based on essential oils may share common antibacterial mechanisms, resulting in comparable effects on eggshell bacterial load and incubation outcomes (Figure 4). The close association observed between *Rosmarinus officinalis* and *Zingiber officinale* essential oils, suggests that these treatments promoted similar responses across both microbiological and incubation variables, which is consistent with evidence indicating that different essential oils can exert antibacterial activity through analogous modes of action, such as disruption of the bacterial cell membrane, increased membrane permeability, and leakage of intracellular constituents [25], which may explain the similarity in responses detected by the clustering procedure.

However, antimicrobial effects are not necessarily uniform among essential oils. The separation of treatment *Cymbopogon flexuosus* essential oil into an independent cluster at higher dissimilarity levels indicates a distinct response, likely associated with differences in chemical composition or concentration. These variations may lead to differences in antimicrobial efficacy and in the interaction between the sanitizer and the eggshell surface, resulting in response patterns that differ from those observed for the other essential oil–based treatments.

In parallel, clustering of formaldehyde and control samples indicates a distinct response pattern compared with that of essential oil–based treatments. Formaldehyde is known to exert its antimicrobial activity through protein cross-linking and nucleic acid damage [26]. However, this mechanism is also associated with toxic effects [27], which have been linked to adverse outcomes during incubation, including impaired embryonic development and reduced hatchability. The later incorporation of grain alcohol into the formaldehyde–control cluster at higher dissimilarity levels further supports the notion that these treatments share closer functional similarities with formaldehyde than with essential oils, albeit with less pronounced effects. Future experiments should incorporate additional control treatments within the same study beyond those evaluated here, such as 70% ethanol, which is widely used as a sanitizing agent. Establishing such comparisons would contribute to a more comprehensive evaluation of emerging sanitizing agents and to the development of improved protocols for commercial poultry hatchery operations, such as the sanitization of hatching eggs.

This study adopts an approach that provides additional insights not explored in the previous study [17]. Unlike the original analysis, the present work applied multivariate statistical techniques to simultaneously integrate microbiological and incubation variables, allowing the identification of interrelationships among these parameters. This approach revealed response patterns that cannot be detected when the variables are evaluated individually, highlighting how the dynamics of microbial contamination interact with incubation performance under different sanitization strategies.

## 4. Conclusions

The multivariate approach applied herein showed clear interdependencies between eggshell contamination, internal bacterial dissemination, embryonic mortality, and hatchability. Although analyses did not detect significant differences for certain incubation performance variables, such as hatchability and embryonic mortality, when evaluated individually, the simultaneous integration of microbiological and incubation variables in the multivariate analyses allowed the treatments to be distinguished based on their combined response profiles. Under the formulations and experimental conditions evaluated, essential oil–based treatments demonstrated a more favorable multivariate balance between microbial control and incubation performance than formaldehyde. The results presented in this study validate the use of multivariate analyses as effective tools for understanding the interactions occurring in the management of hatching eggs, particularly in relation to egg sanitization. These approaches allow a more accurate characterization of sanitization efficacy, supporting the development of alternatives such as essential oils to conventional chemical sanitizers.

## Figures and Tables

**Table 1:** Mean values ± standard deviation of microbiological and incubation variables for different sanitization treatments ^1^.

Variables	Treatments						
	Control	GA	FA	ZOEO	CFEO	ROEO	*p* Value
EWL (%)	12.87 ± 0.95	11.59 ± 0.18	12.82 ± 0.61	12.24 ± 0.61	12.77 ± 1.07	12.34 ± 0.43	ns
CW (g)	45.97 ± 0.67 ^ab^	46.28 ± 0.92 ^a^	44.94 ± 0.42 ^b^	46.32 ± 0.33 ^a^	46.50 ± 0.57 ^a^	46.14 ± 0.31 ^ab^	0.0156
HI (%)	90.36 ± 4.88	92.02 ± 3.30	90.29 ± 2.28	93.48 ± 3.60	93.16 ± 1.06	94.66 ± 2.04	ns
EM (%)	2.98 ± 2.06	1.79 ± 1.54	3.27 ± 1.14	1.79 ± 1.19	1.49 ± 1.14	1.79 ± 2.06	ns
IM (%)	0.00 ± 0.00	0.30 ± 0.60	0.30 ± 0.60	0.60 ± 1.19	0.30 ± 0.60	0.00 ± 0.00	ns
LM (%)	3.57 ± 5.05	5.36 ± 2.06	4.17 ± 2.06	3.27 ± 2.03	3.57 ± 1.68	2.98 ± 1.54	ns
CE (%)	2.08 ± 2.45	0.00 ± 0.00	1.19 ± 1.37	0.30 ± 0.60	0.60 ± 1.19	0.00 ± 0.00	ns
EGM (log_10_ CFU/mL)	1.60 ± 0.30 ^a^	1.13 ± 0.25 ^a^	<1 ^b^	<1 ^b^	<1 ^b^	<1 ^b^	<0.0001
YSM (log_10_ CFU/mL)	2.58 ± 0.39 ^a^	1.66 ± 0.48 ^ab^	1.99 ± 0.10 ^ab^	1.06 ± 0.20 ^b^	1.13 ± 0.99 ^b^	1.00 ± 0.35 ^b^	0.0137

^1^ Source: dos Santos et al. [17]. Abbreviations: Egg weight loss (EWL); chick weight (CW); Hatchability of fertile eggs (HI); early mortality (EM); intermediate mortality (IM); late mortality (LM); number of contaminated eggs (CE); eggshell mesophilic counts (EGM); yolk sac mesophilic counts (YSM). Grain alcohol (GA); formaldehyde (FA); *Zingiber officinale* essential oil (ZOEO); *Cymbopogon flexuosus* essential oil (CFEO); *Rosmarinus officinalis* essential oil (ROEO); not significant (ns). ^a,b^ Different letters in the same row indicate significant differences (*p* < 0.05).

**Table 2:** Correlation between the analyzed variables.

	CW	HI	EM	IM	LM	CE	EGM	YSM
EWL	−0.23	−0.39	0.17	0.13	0.09	0.34	−0.08	−0.29
CW		−0.02	−0.29	0.10	0.19	−0.04	−0.01	−0.24
HI			−0.52 *	−0.29	−0.57 *	−0.41 *	−0.06	−0.34
EM				0.12	−0.22	0.39	0.06	−0.01
IM					−0.00	0.08	−0.26	−0.11
LM						−0.34	0.03	0.29
CE							0.12	0.21
EGM								0.45

Abbreviations: egg weight loss (EWL); chick weight (CW); hatchability of fertile eggs (HI); early mortality (EM); intermediate mortality (IM); late mortality (LM); number of contaminated eggs (CE); eggshell mesophilic counts (EGM); yolk sac mesophilic counts (YSM); * *p* < 0.05.

**Table 3:** Discriminant analysis of the variables responsible for separating the treatments.

Variables	R^2^ Partial	*p* < F	*p* < Lambda	*p* < ASCC
EGM	0.8464	0.0002	0.0002	0.0002
CW	0.6440	0.0263	<0.0001	0.0014
YSM	0.5348	0.1230	<0.0001	0.0050

Abbreviations: Eggshell mesophilic counts (EGM); yolk sac mesophilic counts (YSM); chick weight (CW). Coefficient of determination (R^2^); Tests for multivariate separation of treatments (*p* < Lambda and *p* < ASCC).

## Data Availability

The data are contained within the article.
